# Step Response Characteristics of Polymer/Ceramic Pressure-Sensitive Paint

**DOI:** 10.3390/s150922304

**Published:** 2015-09-03

**Authors:** Anshuman Pandey, James W. Gregory

**Affiliations:** Aerospace Research Center, The Ohio State University, 2300 West Case Road, Columbus, OH 43235, USA; E-Mail: pandey.46@osu.edu

**Keywords:** pressure-sensitive paint (PSP), polymer/ceramic (PC-PSP), step response

## Abstract

Experiments and numerical simulations have been used in this work to understand the step response characteristics of Polymer/Ceramic Pressure-Sensitive Paint (PC-PSP). A recently developed analytical model describing the essential physics in PC-PSP quenching kinetics is used, which includes the effect of both diffusion time scale and luminescent lifetime on the net response of PC-PSP. Step response simulations using this model enables an understanding of the effects of parameters, such as the diffusion coefficient of O_2_ in the polymer/ceramic coating, attenuation of excitation light, ambient luminescent lifetime, sensitivity, and the magnitude and direction of pressure change on the observed response time scales of PC-PSP. It was found that higher diffusion coefficient and greater light attenuation lead to faster response, whereas longer ambient lifetime and larger sensitivity lead to slower response characteristics. Due to the inherent non-linearity of the Stern-Volmer equation, response functions also change with magnitude and direction of the pressure change. Experimental results from a shock tube are presented where the effects of varying the roughness, pressure jump magnitude and luminophore probe have been studied. Model parameters have been varied to obtain a good fit to experimental results and this optimized model is then used to obtain the response time for a step decrease in pressure, an estimate of which is currently not obtainable from experiments.

## 1. Introduction

Pressure-sensitive paint (PSP) is an optical method for determining a quantitative description of surface pressure distribution on aerodynamic bodies. Due to its ability to provide a high-resolution pressure field, PSP has found applications in a myriad of studies [1,2] confirm the ref order through the whole paper for calculating pressure loads, validating CFD results and for inferring flow physics. With the conventional PSP well established as a sensor technology in industries and research labs around the world for steady state testing, over the last decade, research and development in PSP has focused on its improvement for unsteady applications. Improvements in the physical structure of the PSP have led to the development of fast-responding versions of PSP (Fast-PSP) [3,4], which are suitable for measuring pressure fluctuations at frequencies well above 1 kHz. 

**Figure 1:**
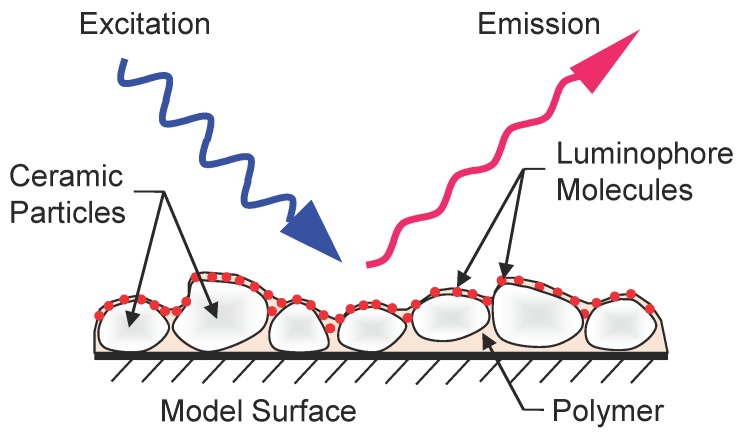
Schematic of PC-PSP.

Polymer/Ceramic PSP (PC-PSP) is a sprayable form of Fast-PSP that can be readily applied on an aerodynamic model before testing. It consists of a polymer/ceramic basecoat, which is first applied on the model as a thin layer and is subsequently used to host the luminophore molecules. The intensity of the excited-state luminescence is modulated based on the concentration of oxygen in the vicinity of the excited luminophores (Figure 1). This dependence of intensity on local pressure is exploited to obtain the pressure information. For improved response characteristics, PC-PSP uses a heavy loading of ceramic particles, which creates voids in the continuum of the polymer/ceramic coating. This leads to improved porosity and larger scattering of excitation light, which decreases the effective thickness of the PC-PSP coating, thereby decreasing the response times to pressure changes. Since PC-PSP imposes no limitation on the model material, it has received wide interest among researchers to characterize unsteady flows.

As PC-PSP is increasingly used to study unsteady problems, it is necessary to ascertain the dynamic characteristics of this sensor technology in order to quantify the limits of its application and to develop compensation methods for applications beyond those limits. Experimental frequency response measurements have shown that at 7–8 kHz, a typical PC-PSP coating has amplitude attenuation of −3 dB and a phase lag of 35° [5]. These studies have also improved the physical understanding PC-PSP quenching dynamics. Unlike conventional and other Fast-PSPs, the thickness of the coating does not affect the response of PC-PSP [6]; rather, the response is due to only a very small top part of the coating (about the roughness of paint) actively participating in the response characteristics. It has been demonstrated [7] that this thickness-independent response is a characteristic of adsorbed-type PSPs where the luminophores are adsorbed onto a pre-coated base layer. This roughness region of the paint coating is easy to maintain during spray coating and coatings can have different thicknesses but have the same roughness and, hence, the same response [5,6]. In contrast to this behavior, a mixed-type PC-PSP with SiO_2_ particles was developed and tested in [8] and its response time was found to be thickness-dependent (as expected for a mixed-type PSP [7]). For adsorbed-type PC-PSP (referred hereafter as only PC-PSP), increasing the roughness improves the response characteristics [5,9] by increasing the diffusion coefficient of O_2_ in the active part of PC-PSP coating and by improving the scattering of the excitation light. Frequency response experiments [6] have also shown that the quenching kinetics are fast enough that the steady-state luminescent lifetime affects the response characteristics of PC-PSP. Attenuation of excitation light also plays a major role in the response characteristics with a larger attenuation leading to faster response due to smaller effective thickness of the paint [5]. Analytical modeling of frequency response [4,10] has also been used in conjunction with these experimental results to gain further understanding of the dynamic response mechanisms. Comparison of simulated frequency response with experimental data has been done [4] to obtain preliminary quantitative estimates of physical parameters like diffusion coefficient and optical depth.

Though a great deal of quantitative information and physical understanding has been achieved from the frequency response studies, the response characteristics of PC-PSP at frequencies above 10 kHz are still not well understood. This has been due to physical limitations in creating repeatable frequency fluctuations at higher frequencies in those studies. To understand the response time scales, step response studies are needed since it subjects the PSP to a much higher effective frequency. Since a shock tube generates a large pressure change within a very short time, it also facilitates the study of the effect of non-linearity on response of PC-PSP. Previously, shock tubes or solenoid-valve driven pressure jump devices have been used to quantify the response times of conventional PSPs [11,12,13] and Anodized-Aluminum PSP (AA-PSP) [14,15,16] and its derivatives [17]. In [18], an earlier version of PC-PSP [19] was tested and its response time was documented as 253 µs using a first order fit (time constant). The physical structure of PC-PSP has undergone further improvements since then through decrease of polymer amount and replacement of Al_2_O_3_ ceramic particles with TiO_2_ particles [20]. This adsorbed-type PC-PSP is a commonly used Fast-PSP formulation (commercially available from Innovative Scientific Solutions, Inc., Dayton, OH, USA), but its response timescales have not been completely quantified.

The objective of the current work is to obtain an estimate of the step response time for this PC-PSP and to understand the physical parameters that affect it. For this purpose, experimental response time measurements for a step increase in pressure are performed in a shock tube and an analytical model (originally developed by Kameda [9] and built upon by Pandey and Gregory [4] for direct comparison with experimental frequency response data) is used to describe the observed response. Model parameters are tailored to provide a best fit to the observed response functions and then this optimized model is used to obtain the response time for a step decrease in pressure, an estimate of which is currently not obtainable from experiments. In order to understand how well the knowledge from limited frequency response studies extrapolates to higher frequencies, the parameters obtained in the current study are compared with those of the previous frequency response study [4]. To begin with, the model is briefly described in the next section along with its numerical implementation. In the subsequent sections, the shock tube setup used to obtain step change in pressure is described and corresponding step response results are presented and discussed with model fits. An estimate for response time of a typical PC-PSP coating for a step decrease in pressure has been obtained from this optimized model. Simulated response from this model is then used to explore the effect of varying physical parameters such as diffusion coefficient, attenuation of excitation light, ambient luminescent lifetime and sensitivity.

## 2. Analytical Model and Numerical Implementation 

A simplified analytical model was developed by Kameda [10], which accounts for the essential physics in PC-PSP quenching kinetics: one-dimensional diffusion of O_2_ into the paint coating and first order population dynamics of excited-state luminophores. The model was further developed by Pandey and Gregory [5] to account for attenuation of excitation light and heterogeneity of the paint layer, and it was tailored to adsorbed-type PC-PSP by using a two-layered structure. This configuration of the model is briefly described here for a homogeneous coating.

Since the thickness of a paint coating is much smaller than the surface area of application, one-dimensional diffusion is assumed to model the permeation of O_2_ molecules over time after a pressure change:

(1) $$\frac{\partial {\left[{\text{O}}_{2}\right]}_{i}}{\partial t}=D\frac{\partial^{2}{\left[{\text{O}}_{2}\right]}_{i}}{\partial x^{2}}$$

**Figure 2:**
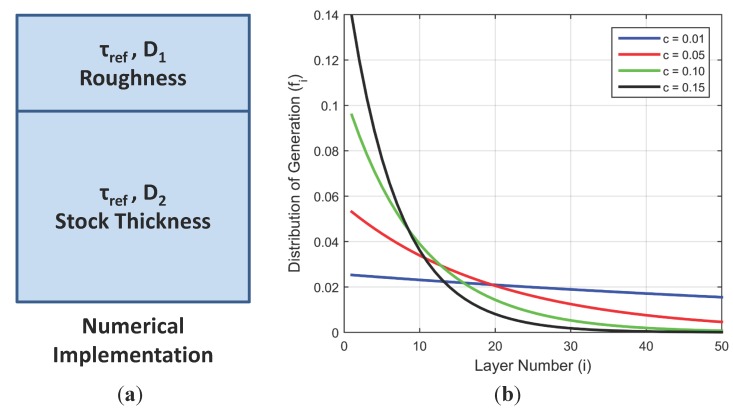
(**a**) Schematic of the two-layered structure used for modelling adsorbed-type PC-PSP; (**b**) Distribution of generation for different values of *c*.

Equation (1) can be solved over a discretized paint layer to obtain the local O_2_ concentration, ${\left[{\text{O}}_{2}\right]}_{i}$, of oxygen in the *i*th layer (of discretization), where *x* is along the depth of the paint and *D* is the diffusion coefficient. For the adsorbed-type PC-PSP, a two-layered structure as shown in Figure 2a is assumed since experimental observations indicate no variation in response with thickness. The bottom layer is assumed to be of a constant diffusion coefficient and is about the thickness of the coating, whereas the top layer corresponds to the final deposition of basecoat which constitutes the surface roughness of the paint. Since this top layer has a much more open structure for rapid diffusion, it is assumed to have a much higher diffusion coefficient (*D*_1_) than the lower layer (*D*_2_).

The first order population conservation equation for the number of excited state luminophores can be reduced (see [5] for details) to the following equation which models the generation of intensity ratio in the *i*th layer:

(2) $$\tau_{ref,i}\frac{d}{dt}\left(\frac{I_{i}\left(t\right)}{I_{ref,i}}\right)+\left(1-B_{i}\left(\frac{{\left[{\text{O}}_{2}\right]}_{i}\left(t\right)}{{\left[{\text{O}}_{2}\right]}_{ref,i}}-1\right)\right)\left(\frac{I_{i}\left(t\right)}{I_{ref,i}}\right)=1$$

where *τ_ref_* is the luminescent lifetime at ambient pressure and $B_{i}=k_{Q}{\left[{\text{O}}_{2}\right]}_{ref,i}/\left(k_{R}+k_{NR}+k_{Q}{\left[{\text{O}}_{2}\right]}_{ref,i}\right)$, is the sensitivity of the *i*th layer. Both of these physical parameters can in general vary over the thickness of the coating, but are assumed to be constant throughout for a homogeneous coating. Given ${\left[{\text{O}}_{2}\right]}_{i}(t)$, Equation (2) can be multiplied with the fraction $f_{i}$ (distribution of incident excitation throughout the paint thickness) and numerically integrated across all layers to obtain the observed intensity response,

(3) $${\left(\frac{I\left(t\right)}{I_{ref}}\right)}_{observed\;}=\;\overset{m}{\underset{i=1}{\sum }}\left(\frac{I_{i}\left(t\right)}{I_{ref,i}}\right)f_{i}$$

Once light is integrated through the thickness of the paint, a static calibration is then used to convert the intensity ratio to measured pressure fluctuation,

(4) $${\left(\frac{P\left(t\right)}{P_{ref}}\right)}_{measured}=\frac{1}{B}\left\{{\left(\frac{I_{ref}}{I\left(t\right)}\right)}_{observed\;}-\left(1-B\right)\right\}$$

(Note that Gregory and Sullivan [21] and Pandey and Gregory [22] have shown that it is important to interpret results based on the indicated pressure, rather than the intensity response, due to the non-linearity of the Stern-Volmer relationship). In the end, this measured pressure ratio is compared to the step change used in the boundary condition of the diffusion equation to obtain the simulated response time. For the case when oxygen diffusion is so fast that it does not play a role in the quenching kinetics, we can analytically integrate Equation (2) using the static calibration to obtain:

(5) $$\frac{I_{i}\left(t\right)}{I_{ref,i}}=\frac{\tau_{final,i}}{\tau_{ref,i}}+\left(\frac{\tau_{initial,i}-\tau_{final,i}}{\tau_{ref,i}}\right)\text{exp}\left(-\frac{t}{\tau_{final,i}}\right)$$

Where $\tau_{initial}$ and $\tau_{final}$ are lifetime values at initial and final pressure, respectively. Using Equation (3), these local intensity ratios can again be integrated throughout the thickness to obtain the observed response; however, since oxygen concentration is the same everywhere, the intensity ratio of every layer is the same as the overall observed intensity ratio—*i*.*e*., integration is not required. It can be seen from Equation (5) that in this limiting case of instantaneous diffusion, the response time scale of PC-PSP is governed by the steady-state luminescent lifetime at the final pressure. 

To numerically implement the above model, Equation (1) is discretized over the thickness of the coating using a finite difference scheme and the interface between the top and bottom layers is modeled as described by Crank [23]. Finer discretization steps were used for the top layer (the active part of the coating): a typical sample of 40 µm thickness and 3 µm roughness is discretized into 2000 layers, of which the top 1000 are assigned to the top 5 µm while the lower 1000 are assigned to the remainder of the thickness. Boundary conditions for the model are a step change in pressure (magnitude and direction of which was varied) at the top and an impermeable wall at the bottom. An initial condition of uniform oxygen concentration (atmospheric pressure) throughout the paint layer is assumed. Once the local oxygen concentration as a function of time is known at each layer, Equation (2) is used to determine the local emission intensity ratio. To obtain the observed intensity ratio from Equation (3), the fraction $f_{i}$ is defined as:

(6) $$f_{i}=\;\frac{e^{-c\left(i-1\right)}}{\sum_{i=1}^{m_{active}}e^{-c\left(i-1\right)}}$$

This fractional contribution of each layer is based on the assumption that attenuation of incident light with depth can be described by the Beer-Lambert Law—*i*.*e*., $I_{x}=e^{-(hf)x}$ where $I_{x}$ is the irradiance at depth *x*, and *hf* is the exponential attenuation factor (described hereafter as the hiding factor). In Equation (6), *c* is the product of hiding factor (*hf*) of the Beer-Lambert Law with the thickness of each layer—*i*.*e*., *c* is dimensionless and *m_active_* is the number of active layers. Figure 2b presents the effect of hiding factor on relative contribution of each paint layer to the overall response. For higher values of *c* (or *hf*), more generation of excited state luminophores happens in upper layers (closer to the surface), and hence those layers play a larger role in the observed dynamics of PC-PSP as per Equation (3). 

## 3. Experimental Framework

The experimental results presented in this study were conducted with samples prepared using the standard formulation of PC-PSP used by many researchers [3,4]. Readers are referred to [5] for details on the sample preparation procedure and techniques for thickness and roughness measurement. In this work, the effect of luminophore was also explored by comparing the response of tris-(bathophenanthroline) ruthenium (II) chloride (RuDPP) (from GFS Chemicals) with that of platinum tetra (pentafluorophenyl) porphyrin (PtTFPP) (from Frontier Scientific). In each case, the luminophore solution was prepared by dissolving 0.3 mg of luminophore per mL of methanol before spraying onto the polymer/ceramic basecoat.

Static calibration of the samples was performed using a calibration chamber which can achieve set points in temperature and pressure independently of one another. A pulsed LED array (400 nm for PtTFPP and 460 nm for RuDPP) (LM2x-DMHP from ISSI, Dayton, OH, USA) was used to obtain lifetime scans at steps of 20.6 kPa from 20.6 kPa to 206 kPa while the temperature was fixed at 298 K. Five hundred decay scans were averaged and were fit with a single exponential function, using data from the end of the excitation pulse until approximately the 90% emission decay point (to avoid the noise floor).

**Figure 3:**
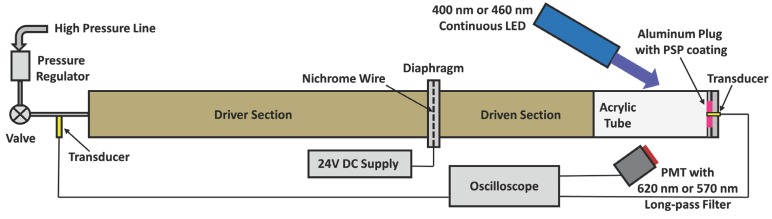
Shock tube setup.

To validate the numerical simulations, experimental step response data for PC-PSP is required. The shock tube is a preferred tool in Fast-PSP research due to its ability to create a step change of any magnitude in less than 1 µs. However, shock tubes can only create a pressure increase. Another limitation is the production of a large step increase in temperature along with pressure increase, although it has been observed that the increase in sample temperature over the time scale of pressure response is negligible [14,15]. A schematic diagram of the shock tube setup used in this work is shown in Figure 3. The PSP was mounted on the end wall in order to exploit the combined power of initial and reflected shock to generate a large change in pressure, and to increase the amount of light collected for high signal-to-noise ratio (SNR).

**Figure 4:**
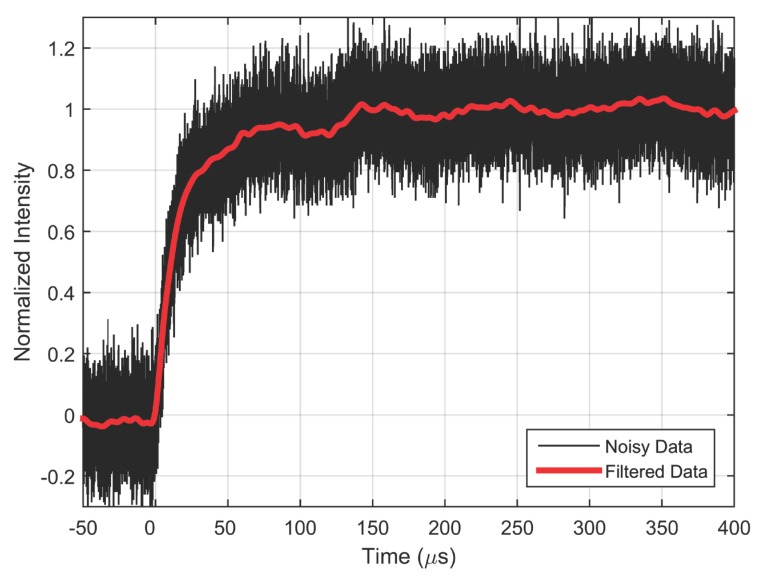
Normalized PMT signal and the corresponding filtered data.

The shock tube was constructed of a 5 cm inlet diameter PVC tube with driver and driven sections measuring 1.83 m and 0.75 m long, respectively. An acrylic tube was used to provide optical access to the setup, with a PC-PSP coated aluminum plug placed at its end. The driver section was pressurized using air from a high pressure line while a transducer monitored the pressure rise. The driven section was maintained at ambient pressure until rupture of the diaphragm. Once the desired driver section pressure was reached, a valve was closed to isolate the shock tube. The diaphragm which separates the high pressure driver section from the driven section was constructed of a 0.1 mm acetate sheet which was burst in a controlled manner with a heated Nichrome wire. A continuous 3-W LED array (400 nm for PtTFPP and 460 nm for RuDPP) (LM2x-DM from ISSI, Dayton, OH, USA) excited the PC-PSP samples. Paint emission was captured using a photomultiplier tube (PMT) (Hamamatsu h9505-03) fitted with a long-pass filter (620 nm for PtTFPP and 570 nm for RuDPP) to block excitation light. A 300 Ω resistor was used to convert the current output of the PMT to voltage, with the value of the resistor selected to ensure that the dynamic response of the measurement system does not affect the measured response time of PC-PSP. An oscilloscope (Lecroy Waverunner 44Xi) captured 10^5^ instances of the PMT output at a sample rate of 2 GHz, and was triggered by a transducer collocated at the end of the shock tube. Post processing of the acquired time scans involved a low-pass digital FIR filter with passband frequency of 1 MHz. Figure 4 shows the normalized recorded PMT data along with the filtered data which was used for comparison with model. All the figures have been normalized using:

(7) $$I_{n}(t)=\frac{I(t)-I_{initial}}{I_{final}-I_{initial}}\text{ ; }P_{n}(t)=\frac{P(t)-P_{initial}}{P_{final}-P_{initial}}$$

As suggested by Sakaue *et al*. [16], normalized pressure can also be obtained by:

(8) $$P_{n}(t)=\frac{\left(I_{ref}/I\right)-{\left(I_{ref}/I\right)}_{initial}}{{\left(I_{ref}/I\right)}_{final}-{\left(I_{ref}/I\right)}_{initial}}$$

which does not require explicit application of the static calibration for conversion of intensity ratio to pressure ratio. It can be observed that the shock tube setup produces a clean initial rise in pressure but has some fluctuations in the steady state region. These fluctuations (of the order of 5%) are typically found in shock tube results [17]. In this paper, the response time is defined as the time taken for intensity or pressure to reach 90% of the final value ($t_{90}$).

## 4. Results and Discussion

The primary objective of this work is to estimate the step response time of PC-PSP and to study the paint response at higher frequencies. To this end, step response measurements for a typical sample are presented, along with the simulated step response obtained from the model fit to the experimental data. The model fit parameters obtained from this work are then compared with parameters obtained from a previous frequency response study [5]. Additionally shown are the experimental effects of varying the final pressure and the luminophore, in order to validate the model. The optimized model is then used to obtain the response time for a step decrease in pressure and for understanding the effect of various physical parameters via simulations.

### 4.1. Experimental Results and Determination of Model Parameters

**Figure 5:**
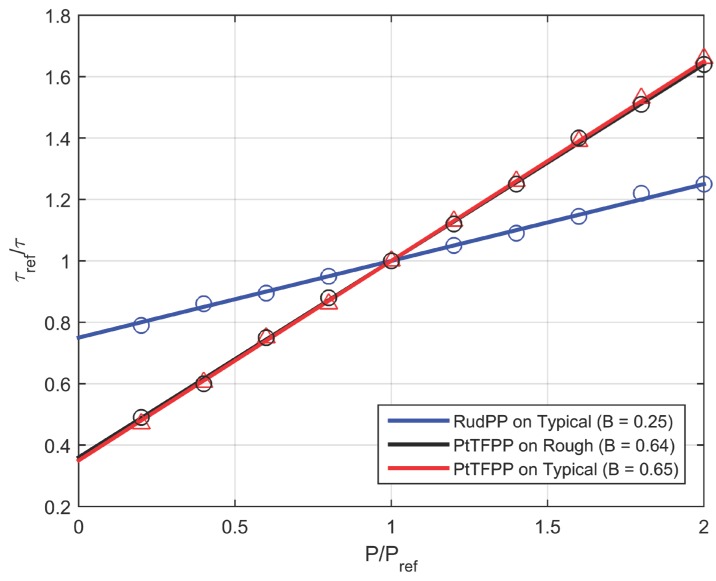
Static Calibration where $P_{ref}$ and $\tau_{ref}$ correspond to the reference (ambient) conditions.

Measured values of paint sample characteristics for typical and rough coatings are listed in Table 1 and static calibrations for the samples are shown in Figure 5. Static calibration as obtained from the exponential functions describing the lifetime decay at different pressures is usually presented in this “linear” form by taking the reciprocal of the estimated lifetime values. Ambient condition ($P_{ref}$ = 103 kPa and T = 298 K) was used as reference and for normalizing, lifetime value at this reference condition ($\tau_{ref}$) was used. It can be observed that paint sensitivity, defined in Equation (2) as the slope of the static calibration, is only slightly affected by the roughness of the coating. However, the lifetime at ambient pressure is smaller for a rougher sample since a more open structure provides for a higher quenching rate by ambient O_2_. Uncertainty in these measurements has been discussed in [5].

**Table 1:** PtTFPP sample characteristics.

Sample	Thickness	Roughness	$\tau_{ref}$	Sensitivity
Typical	44.12 μm	3.16 μm	7.30 µs	0.65
Rough	104.2 μm	23.85 μm	6.75 µs	0.64

**Figure 6:**
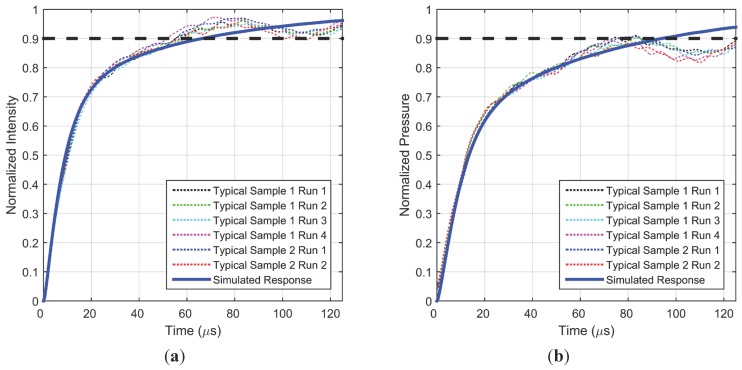
Measured and simulated intensity (**a**) and pressure (**b**) step response for a typical PC-PSP coating over different runs and samples.

Figure 6 shows the normalized step response (intensity and pressure response) for two representative samples with typical roughness. The shock tube was run with a driven pressure of 213 kPa, which subjected the sample to a step change in pressure from 101.3 kPa to 205.6 kPa. The repeatability of the setup is demonstrated by the similar response over four separate runs for a typical sample. Repeatability across samples is also demonstrated by this figure as two similarly prepared samples have nearly the same step response characteristics. Fluctuations in the steady-state region are of the same order as those shown in Figure 4, but appear more pronounced due to the zoomed-in time window. Additionally shown in Figure 6 is the simulated response for this step change in pressure, with the model fit parameters listed in Table 2. With these parameters, the simulated response produces a very good match to the initial rise characteristics of the experimental results. During simulations, the hiding factor was fixed at the previously modeled value of 1.67 × 10^6^ m^–1^, while the diffusivity of the top layer was allowed to vary. The best fit value of the diffusion coefficient is well within an order of magnitude of the value obtained from previous frequency response work [5] (compared in Table 2), thus providing confidence in the modelling effort. The lifetime value input into the model was slightly different from the previous work; however it is known from Sugimoto *et al*. [6] and modeling efforts by Pandey and Gregory [5] that such small a difference will not produce any observable change in response time. Static calibration was used to convert the simulated and experimental intensities to pressure which are shown in normalized form. While the PC-PSP intensity response observed in Figure 5 is only 70 µs, the pressure response is about 100 µs. These step response results are comparable to, but longer than, the step response of PtTFPP on AA-PSP which was documented as 50 µs for 90% rise [16]. 

**Figure 7:**
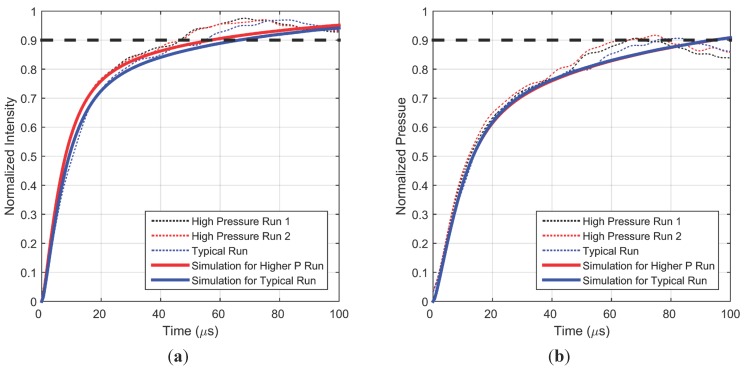
Measured and simulated intensity (**a**) and pressure (**b**) step response for higher final pressure.

In order to validate the parameter values obtained by the model fit to the experimental data, an experiment with a higher pressure jump was conducted to study the effect of non-linearity in intensity response. The driver section was charged to 275.8 kPa which produced a step change from 101.3 kPa to 257.4 kPa (more than 50 kPa higher than a typical run) at the PC-PSP location. As shown in Figure 7, a faster intensity response is obtained for this larger pressure change due to the lower luminescent lifetime existing at higher pressure. Larger fluctuations are also observed in the steady state region but are again repeatable as can be seen from the two high pressure runs. The time window has also been zoomed in for improved visualization of the fine difference in intensity response times. For simulating this higher pressure run using the model, all the parameters were fixed at the same values as those found by fitting to earlier experimental data, with only the final pressure in the diffusion equation being changed to this higher value. The simulations continue to correspond very well with the experimental measurements. Figure 7b shows that calibrated pressure data shows much less of a difference in response at different pressure conditions, compared to the intensity response data in Figure 7a.

**Figure 8:**
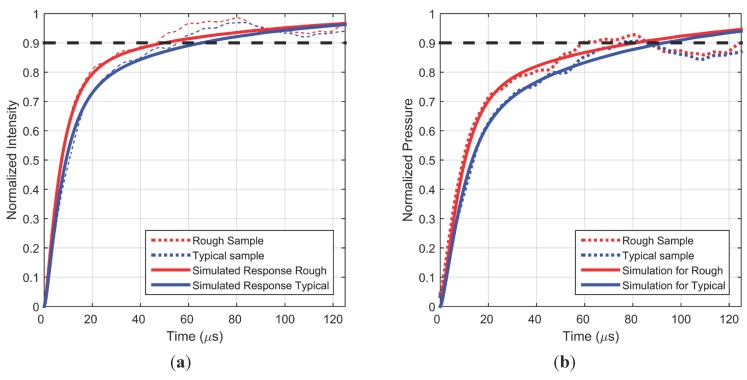
Measured and simulated intensity (**a**) and pressure (**b**) step responses for a typical sample and a rough sample.

The roughness of the PC-PSP basecoat has been found to affect the frequency response of the paint coating by improving the permeability of O_2_ [5,9]. In order to confirm this at higher frequencies, a rough sample was evaluated in the shock tube setup (see Table 1 for roughness values). Figure 8 compares the step response for typical and rough samples as both samples are subjected to a pressure change of the same magnitude. The intensity response of the rough sample is much faster than that of a typical sample. The simulated response found from the best fit to the rough sample data is also shown. The same model fit methodology was used: lifetime and hiding factor were fixed at the values known from the frequency response study, while the diffusion coefficient was varied to obtain the best fit. This exercise gives an estimate of the largest uncertainty in the knowledge of this physical parameter. Table 2 lists the fit parameters, and it can be seen that diffusion coefficient is again well within an order of magnitude of the previous study. Figure 8b shows that this fast intensity response of a rough coating also translates into a 20% faster pressure response, since the pressure change and sensitivity are the same as in the typical case. This improvement in response time with increased surface roughness can be exploited in applications where the impact of surface roughness on the flow is negligible.

**Figure 9:**
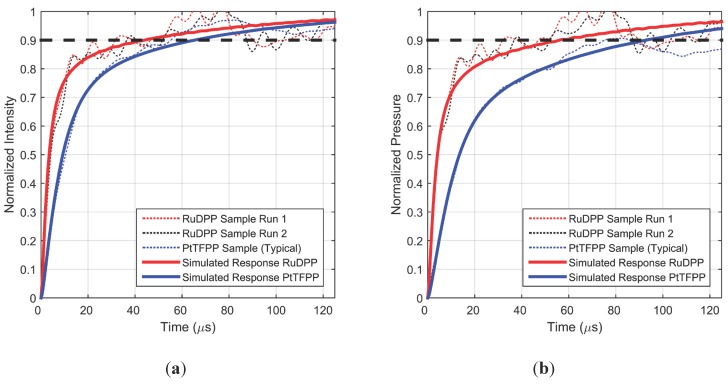
Measured and simulated intensity (**a**) and pressure (**b**) step response for different luminophores.

Sugimoto *et al*. [6] showed that a faster frequency response was obtained by using a RuDPP (dissolved in Dichloromethane) based PC-PSP instead of a PtTFPP based PC-PSP. It was suggested that this is due to the smaller ambient pressure lifetime of the RuDPP in PC-PSP. To further understand this phenomenon, a RuDPP (dissolved in Methanol) based sample was prepared by spraying onto a typical PC-PSP basecoat for direct comparison of the effect of luminophore. Since the solvent used in this comparison is the same and typical coatings have similar roughness values [4], it is assumed that RuDPP based PC-PSP will have the same diffusion coefficient as a typical PtTFFP based PC-PSP. Static calibration was conducted on this RuDPP based sample (Figure 5) and the sensitivity was found to be 0.25 and ambient lifetime ($\tau_{ref}$) was recorded as 2 µs. Experimental step response data is shown in Figure 9 and it can be seen that, like the frequency response measurements reported in [5], RuDPP based PC-PSP produces a much faster step intensity response compared to the PtTFPP response. Data is noisier due to the smaller SNR over this pressure change as a result of the lower sensitivity. This higher frequency noise is different from the steady-state fluctuations discussed earlier and is also commonly found in shock tube results [16]. A simulated response was fit to this data with the recorded value of lifetime and the assumption that diffusivity is same as that of a typical PtTFPP based sample. A hiding factor of 2.9 × 10^6^ m^–1^ was found from curve fitting, which suggests that light attenuation is much higher in the presence of RuDPP. The RuDPP based PC-PSP step response provides about 50% improvement in the pressure response time scale, which can be exploited in studies where the magnitude of pressure change is high enough to provide a good SNR.

**Table 2:** Model parameters for curve fits.

Sample	Parameter	Step Response Study	Frequency Response Study [5]
Typical roughness with PtTFPP	Lifetime (µs)	7.30	7.04
	*D*_1_ (m^2^/s)	5 × 10^–8^	9.78 × 10^−8^
	Hiding Factor (1/m)	1.67 × 10^6^	1.67 × 10^6^
Larger roughness with PtTFPP	Lifetime (µs)	6.75	6.75
	*D*_1_ (m^2^/s)	2.5 × 10^–6^	7.51 × 10^−6^
	Hiding Factor (1/m)	3.13 × 10^5^	3.13 × 10^5^
Typical roughness with RuDPP	Lifetime (µs)	2.00	N/A
	*D*_1_ (m^2^/s)	5 × 10^–8^	N/A
	Hiding Factor (1/m)	2.90 × 10^6^	N/A

### 4.2. Numerical Simulations 

#### 4.2.1. Estimate of Response Time 

**Figure 10:**
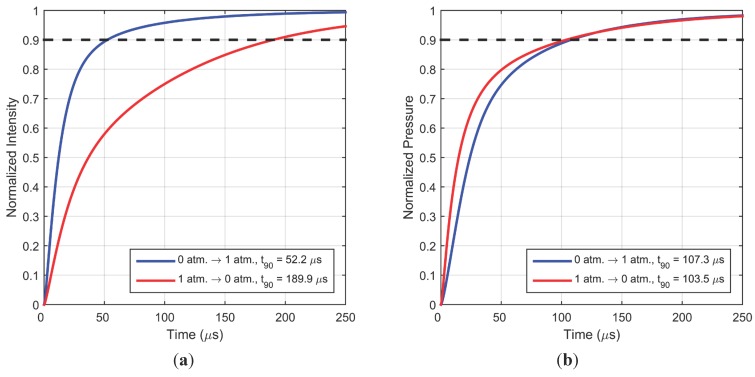
(**a**) Simulated intensity response; (**b**) Simulated pressure response for a typical sample for a step change in pressure between vacuum and 1 atm.

Simulations using the analytical model described in Section 2 were compared with experimental measurements in Section 4.1 and optimized curve fitting was used to estimate the value of diffusion coefficient and hiding factor of typical PC-PSP coatings (Table 2). Diffusivity of the top layer was found to be 5 × 10^–8^ m^2^/s, with the lower layer assumed to have a value 10^3^ times smaller. The hiding factor for violet excitation was found to be 1.67 × 10^6^ m^–1^. An active thickness the same as the roughness of the paint was found to be sufficient for a good curve fit. For a typical sample, an ambient pressure lifetime of 7.30 µs and sensitivity of 0.65 were observed as listed in Table 1. These parameters are now used to estimate the response to a step increase and corresponding step decrease for a pressure change between vacuum and 1 atm. A very important use of this optimized model is the determination of PSP response to step decrease in pressure, since it is very difficult to create a carefully controlled experimental step decrease in pressure. Figure 10 shows that a pressure increase has an intensity response over three times faster than that for a pressure decrease of the same magnitude. This arises from longer relaxation time (lifetime) of excited state molecules at lower pressure. However, non-intuitively, this difference in response times does not persist when static calibration is used to convert the intensity response to pressure. This opposite behavior of intensity and pressure response is due to the inherent non-linearity in the Stern-Volmer relationship which produces a higher intensity change for the same pressure change if the mean is at a lower pressure. In Figure 10b, the simulated response for pressure decrease shows a faster component initially but slows down later with almost similar times to reach 90% of the final value. These findings are in general agreement with the modeling work of Gregory and Sullivan [21], although the fidelity of the model presented here is much higher.

**Figure 11:**
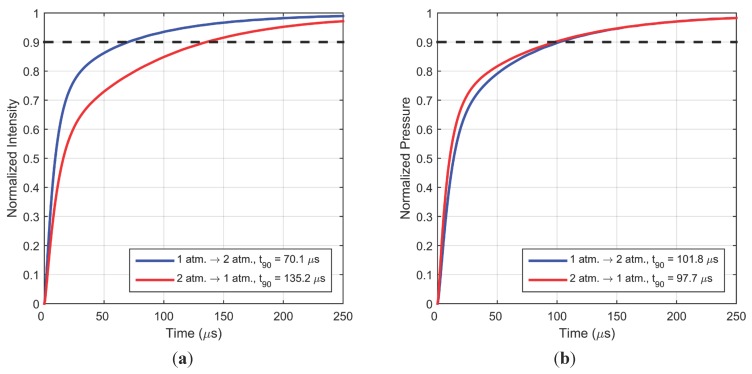
(**a**) Simulated intensity response; (**b**) Simulated pressure response for a typical sample for a step change in pressure between 1 atm. and 2 atm.

Corresponding calculations for a different pressure difference, 1 atm. to/from 2 atm., are shown in Figure 11. The trends observed here are similar to Figure 10: the intensity response in nearly twice as fast for a pressure increase; however the 90% intensity response times are slower for the pressure increase, and faster for the pressure decrease, compared to the values observed in Figure 9. Trends for pressure response are also similar for this pressure difference but the difference between step response of increase and decrease is much less. From these two simulations the 90% response time for step increase and decrease is estimated to be about 102 µs and 98 µs, respectively. This is slightly slower than the previously suggested [3] first-order time constant (for pressure response) of 25 µs, which would give a 90% response time of about 60 µs. 

#### 4.2.2. Origin of Differences in Intensity and Pressure Response

**Figure 12:**
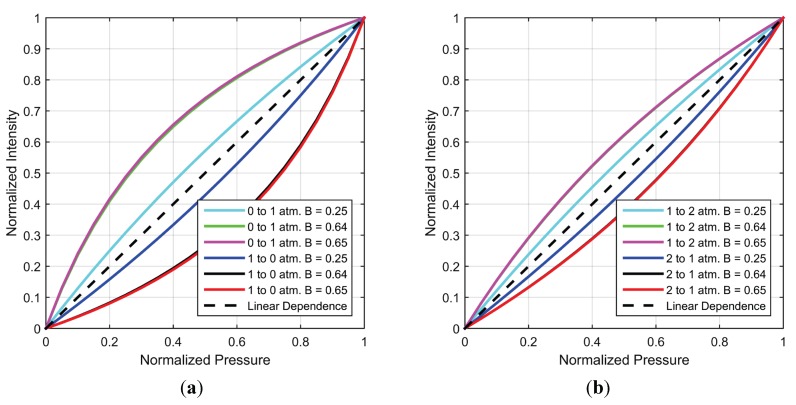
Normalized Intensity *vs.* Normalized Pressure for a pressure jump between (**a**) 0 and 1 atm. and (**b**) 1 atm. and 2 atm.

As observed in the previous sections from experimental data and simulations, translating the intensity response to pressure leads to large differences in response time estimates. This difference in response times arises due to the non-linear dependence of pressure on intensity. As described earlier, taking the reciprocal of experimental lifetime values results in the ‘linear’ static calibrations (Figure 5) which suggests a hyperbolic dependence between pressure and intensity (or lifetime). Further understanding can be gained by using Equation (7) and static calibration data to compare the normalized intensity and pressure as shown in Figure 12. Additionally shown is the hypothetical case of a linear dependence. It should be noted that Figure 12 is independent of the rate of pressure change. 

It can be seen that sensitivity is also a measure of non-linearity such that a larger variation from the one-to-one correspondence between pressure and intensity exists for larger sensitivity values. The hyperbolic nature of the non-linearity is exhibited in the form of larger excursion (from linear dependence) at lower pressure (a) than at higher pressure (b). This explains the larger difference in intensity and pressure response times in Figure 10 as compared to Figure 11. Another affect of hyperbolic dependence is that a given fractional change in intensity signifies a larger fractional change in pressure for pressure decrease than increase. 

#### 4.2.3. Effects of Parameters 

**Figure 13:**
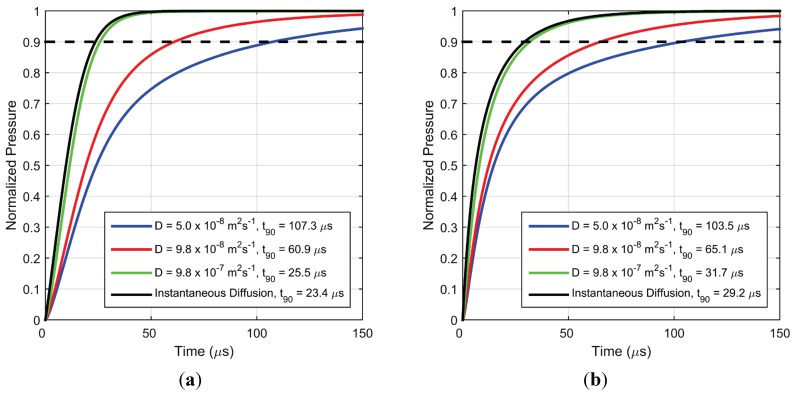
Simulated pressure response for (**a**) step increase in pressure from vacuum to 1 atm. and (**b**) step decrease in pressure from 1 atm. to vacuum, for different values of diffusion coefficient.

In this section, numerical simulations have been used to understand the effect of the various physical parameters in the model on PC-PSP dynamic response. In each case, only one of the parameters is changed while the rest of the parameters are kept fixed at the values described in the previous section. A pressure difference of vacuum to/from 1 atm. was used for this study. Figure 13a,b show the effect of diffusion coefficient of the paint coating for pressure increase and decrease, respectively. Only the diffusivity of the top layer is changed with the lower layer assumed to have a value 10^3^ times higher than the value of the top layer. Previous studies [5,9], as well as the results presented in Figure 7, demonstrate that higher surface roughness improves the quenching kinetics since it provides a more open structure for oxygen diffusion. The trends here are similar to the experimental observations—a larger value of diffusion coefficient leads to shorter response time. The red curve is the expected response time with the parameters from the frequency response study [5] (*D* = 9.78 × 10^–8^ m^2^/s), while the blue curve corresponds to the fit parameters found in the current study (*D* = 5 × 10^–8^ m^2^/s). Although the two response curves are reasonably close, with the higher diffusion coefficient indicating a faster step response, the discrepancy may arise due to the uncertainty in the diffusion coefficient measured by the previous frequency response study [5] where the range of frequencies that could be produced was limited. Also shown is the limiting case when the diffusion is instantaneous (from Equation (5)). This limiting curve is a pure exponential and its response time is governed by the luminescent lifetime at final pressure ($\tau_{final}$). 

**Figure 14:**
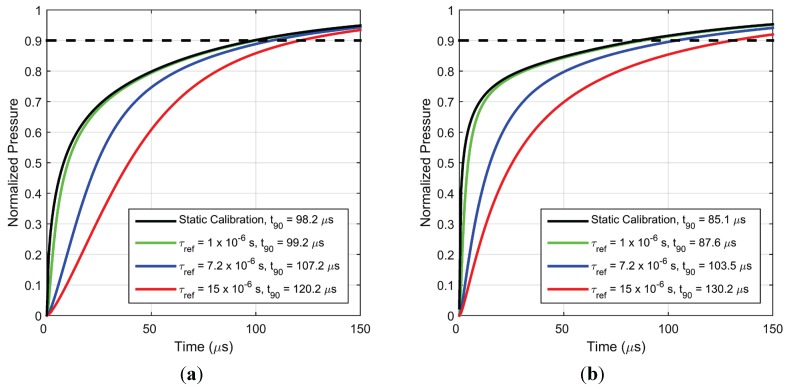
Simulated pressure response for (**a**) step increase in pressure from vacuum to 1 atm. and (**b**) step decrease in pressure from 1 atm. to vacuum, for different values of ambient lifetime.

Figure 14a,b show the effect of lifetime at ambient pressure on the response time scale of PC-PSP for pressure increase and decrease, respectively. It can be inferred that diffusion in PC-PSP is fast enough that lifetime has an effect on the quenching dynamics such that a smaller lifetime would exhibit a smaller response time. These simulations also suggest that a faster responding PC-PSP can be obtained by using a luminophore whose ambient lifetime is smaller. The green curve (for a lifetime about an order smaller than PtTFPP in PC-PSP) shows that considerable improvements in response times scales can be achieved, provided other parameters remain fixed. For an even shorter lifetime, the dynamic response is limited by diffusion (black curve) and can be described by a diffusion-only model, as previously shown in [21]. In this limiting case, the transient term in Equation (2) is dropped and the resulting static calibration equation is directly used to obtain the local emission intensity generation from local oxygen concentration.

**Figure 15:**
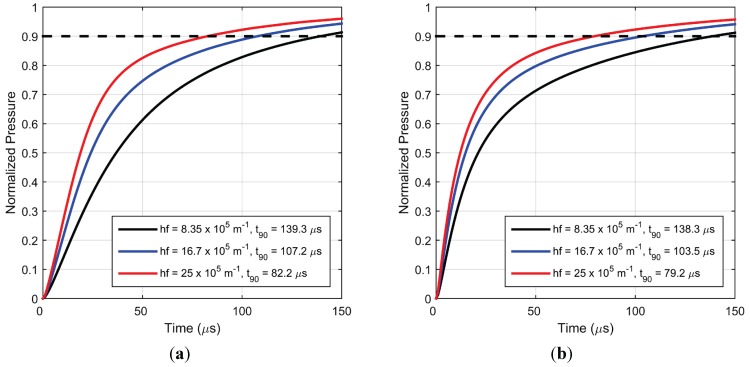
Simulated pressure response for (**a**) step increase in pressure from vacuum to 1 atm. and (**b**) step decrease in pressure from 1 atm. to vacuum, for different values of hiding factor.

As described in the Section 2, attenuation of excitation light due to absorption by luminophores and scattering by TiO_2_ particles is modelled as an exponential decay of incident light. It was shown in Figure 2 that increasing the hiding factor increases the fractional contribution of upper layers to the overall dynamic response of the coating. Since only these upper layers, which are closer to the imposed time-varying surface pressure boundary condition, actively contribute to the dynamic response, a higher value of hiding factor improves the response characteristics of the paint. This behavior was also shown in the previous frequency response simulations in [5]. From Figure 15a,b, it can be inferred that this also holds at higher frequencies and for larger pressure fluctuations. In fact, heavy attenuation of excitation light along with excellent porosity of the roughness region of a PC-PSP coating is the primary reason for its excellent pressure response characteristics. 

**Figure 16:**
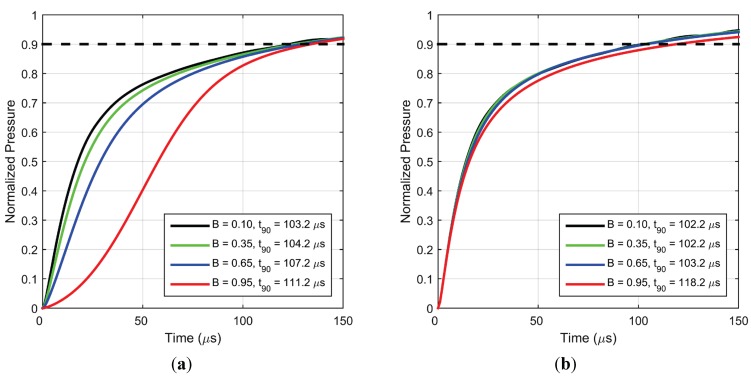
Simulated pressure response for (**a**) step increase in pressure from vacuum to 1 atm. and (**b**) step decrease in pressure from 1 atm. to vacuum, for different values of sensitivity.

The effect of paint sensitivity could not be effectively explored in the previous frequency response studies [5] since small pressure fluctuations (of the order of 1–2 kPa) led to near-linear response of the Stern-Volmer equation. However for large pressure fluctuations (of the order of 1 atm.) as found in a shock tube, non-linearity of the response becomes significant. Figure 16a,b) shows the effects of sensitivity on the response time scales of PC-PSP for a pressure increase and decrease, respectively. If the rest of the parameters (including luminescent lifetime) remain unchanged, it can be deduced that a more sensitive paint will have a slower response time. For a pressure increase, a high value of sensitivity leads to a much slower initial response (red curve in Figure 16a); however at longer times the response is similar. On the other hand, the initial response is comparable for pressure decrease (red curve in Figure 16b) but becomes slower for larger times. Additionally, a larger value of sensitivity produces an even greater difference between the intensity and pressure response characteristics due to more pronounced non-linearity as described in Section 4.2.2. 

## 5. Conclusions

The response times to step changes in pressure have been estimated for typical polymer/ceramic PSPs based on PtTFPP and RuDPP using experiments and numerical simulations. Parameters of the PC-PSP dynamic model (diffusion coefficient and hiding factor) were varied to obtain a good fit to measured step rise data from a shock tube experiment. The model was then used to estimate the response time for a step decrease in pressure which cannot currently be obtained experimentally. Due to the non-linear (hyperbolic) nature of the Stern-Volmer equation, a step decrease in pressure has a shorter response time than a corresponding step increase across same pressure difference. Two important findings of this study are: (1) the dynamic response of PSP samples should be compared across the same pressure change and (2) the intensity response is not the same as the pressure response. Model parameters from the current work were also compared with those obtained from a previous limited frequency response study and this comparison provides a more confident bound on the estimates of diffusion coefficient. Although the values reported in this study were comparable and within an order of magnitude of the previous study [5], the difference may be attributed to the limited frequencies over which the measurements were conducted in the earlier work. The diffusion coefficient for the top active layer (the roughness region) of a typical PC-PSP can now be reported with a high level of confidence to be between 5 × 10^−8^ and 1 × 10^−7^ m^2^/s.
